# Pressure-Sensitive Insoles for Real-Time Gait-Related Applications

**DOI:** 10.3390/s20051448

**Published:** 2020-03-06

**Authors:** Elena Martini, Tommaso Fiumalbi, Filippo Dell’Agnello, Zoran Ivanić, Marko Munih, Nicola Vitiello, Simona Crea

**Affiliations:** 1 The BioRobotics Institute, Scuola Superiore Sant’Anna, 56127 Pisa, Italy; tommaso.fiumalbi@santannapisa.it (T.F.); nicola.vitiello@santannapisa.it (N.V.); simona.crea@santannapisa.it (S.C.); 2 Faculty of Electrical Engineering, University of Ljubljana, 1000 Ljubljana, Slovenia; zoran.ivanic@robo.fe.uni-lj.si (Z.I.); marko.munih@robo.fe.uni-lj.si (M.M.); 3 IRCCS Fondazione Don Carlo Gnocchi, 20148 Milan, Italy; 4 Department of Excellence in Robotics & AI, Piazza Martiri della Libertà, 33–56127 Pisa, Italy

**Keywords:** optoelectronic sensors, wearable sensors, sensorized insole, plantar pressure distribution, real-time gait monitoring, robot control

## Abstract

Wearable robotic devices require sensors and algorithms that can recognize the user state in real-time, in order to provide synergistic action with the body. For devices intended for locomotion-related applications, shoe-embedded sensors are a common and convenient choice, potentially advantageous for performing gait assessment in real-world environments. In this work, we present the development of a pair of pressure-sensitive insoles based on optoelectronic sensors for the real-time estimation of temporal gait parameters. The new design makes use of a simplified sensor configuration that preserves the time accuracy of gait event detection relative to previous prototypes. The system has been assessed relatively to a commercial force plate recording the vertical component of the ground reaction force (vGRF) and the coordinate of the center of pressure along the so-called progression or antero-posterior plane (CoP_AP_) in ten healthy participants during ground-level walking at two speeds. The insoles showed overall median absolute errors (MAE) of 0.06 (0.02) s and 0.04 (0.02) s for heel-strike and toe-off recognition, respectively. Moreover, they enabled reasonably accurate estimations of the stance phase duration (2.02 (2.03) % error) and CoP_AP_ profiles (Pearson correlation coefficient with force platform ρCoP = 0.96 (0.02)), whereas the correlation with vGRF measured by the force plate was lower than that obtained with the previous prototype (ρvGRF = 0.47 (0.20)). These results confirm the suitability of the insoles for online sensing purposes such as timely gait phase estimation and discrete event recognition.

## 1. Introduction

Portable plantar pressure measurement systems have gained popularity in recent years. Initially developed for extending clinical gait assessments to community and real-world environments (beyond clinical and laboratory settings), in-shoe sensor technologies for plantar pressure analysis have lately found new applications outside the clinical domain. Specifically, the proliferation of consumer-grade ‘smart wearables’ has further propelled the development of commercial in-shoe devices to assess health and wellness-related mobility parameters in activities of daily living [1,2,3,4]. In addition to stand-alone sensing applications, portable sensing technologies for plantar pressure measurement have been incorporated in the development of wearable robots.

Robotic prostheses and orthoses require integrated or minimally-encumbering sensors to provide reliable measurements of biomechanical variables, which, in turn, serve as inputs for the control algorithms that run the platforms such as for real-time gait phase estimations or to detect the movement intentions of the user. For example, to control lower-limb prostheses, a timely estimation of the stance/swing gait phases is critical. In fact, the information on the current gait phase can trigger different controllers and thus, a precise detection of foot contact with the ground is crucial for safe and correct functioning of prostheses [5]. Similarly, biofeedback devices need real-time gait-phase estimation in order to provide consistent functional electric stimulation (FES) or active sensory feedback to the users [6,7,8]. Real-time information on plantar pressure distribution can also be exploited to detect the movement intentions of the user and automatically adapt the control action of robotic assistive devices such as prostheses and orthoses to different locomotion tasks (e.g., walking up or down slopes or climbing stairs) [9,10]. Moreover, precise information on the gait phase allows for the estimation of temporal gait indicators that are important for assessment and diagnosis. Gait symmetry, for example, can be used to compare gait performance with different prostheses (and thus rate different prostheses) in the case of amputees, or to evaluate the outcome of rehabilitation programs in pathologies exhibiting gait asymmetries [5]. With diagnostics purposes, machine learning techniques have exploited features extracted from temporal gait parameters to distinguish between different neurodegenerative diseases [11].

The design requirements of in-shoe sensing devices whose primary intended use deals with the real-time control of artificial limbs, orthoses, or biofeedback devices, reflect different priorities than the ones demanded for gait analysis applications. In the latter case, the accuracy and *precision* of the measured gait variables are the parameters of primary importance, whereas for online applications with assistive robotics, *sensor responsivity* is the fundamental indicator to ensure a prompt real-time detection of specific biomechanically-relevant gait events (i.e., typically the heel-strike and toe-off events), hence a timely and synchronous action of the linked wearable device [12,13,14]. Sensory apparatus designs should also consider the richness of the sensory information: multiple sensor signals as well as information related to the interaction with the external environment are necessary to develop sophisticated movement intention detection algorithms that are capable of classifying and even predicting the upcoming locomotion mode. While simple foot switches make it easy to detect specific gait-related events, their on–off response behavior does not allow the extraction of quantitative information about the pressure distribution on the plantar area [6,15,16]. The same limitation applies to foot-mounted inertial measurement units (IMUs), which can provide estimates of ground contact time and other kinematic information (typically in concert with other units on additional limb segments), but cannot sense plantar pressure [17,18,19]. *Reliability* of the measurements is another essential performance indicator: any under-foot pressure-sensing technology must be robust against environmental factors that change throughout the day or prolonged use. Indeed, changes of in-shoe temperature and humidity can lead to measurement drift, which requires the user to execute frequent system re-calibrations, thus making the use of wearable robots impractical [12,20,21,22,23]. Finally, *cost* is a very important factor that may limit the adoption of insoles in applications with wearable robots; hence, even though several emerging technologies are targeting this requirement [24,25,26], the most established insoles on the market, to the author’s knowledge, have never been integrated or tested within robotic devices [2,3,20].

In previous studies, our team demonstrated that pressure-sensitive insoles capitalizing on optoelectronic transduction could estimate the real-time ground-reaction force with fair accuracy [27] and could be effectively exploited for gait segmentation to provide contextual, phase-based sensory feedback [28,29]. This sensing technology [30,31] has been shown to be suitably cost-effective and exhibits further advantages for daily-base use such as immunity to temperature variations and unnecessary calibration procedures [27].

In this study, we present a novel prototype of an in-shoe optical sensor technology for plantar pressure monitoring, optimized for the real-time control of lower-limb wearable robots. The aim of the novel device was to obtain a simplified version of the one presented in [27], with the goal to reduce the number of sensors, and in turn, the overall system complexity and manufacturing costs, still providing a timely detection of the foot-contact events and preserving qualitative information about the antero-posterior weight distribution over the feet. Time accuracy would deem our device comparable—in terms of reliability—with the most commonly adopted solutions for real-time control purposes, yet capable of providing richer online information on plantar pressure. Along with the presentation of the device, the results of verification tests are reported. The primary objective of these tests was to assess the performance of the new prototype for online gait cycle segmentation, given its foreseen application as sensors for controlling lower-limb orthoses or prostheses. Furthermore, its ability to accurately track the temporal evolution of the foot-ground interaction forces was evaluated as richer information that the insoles could valuably provide for further gait assessment purposes.

## 2. Materials and Methods

### 2.1. System Architecture

**Figure 1:**
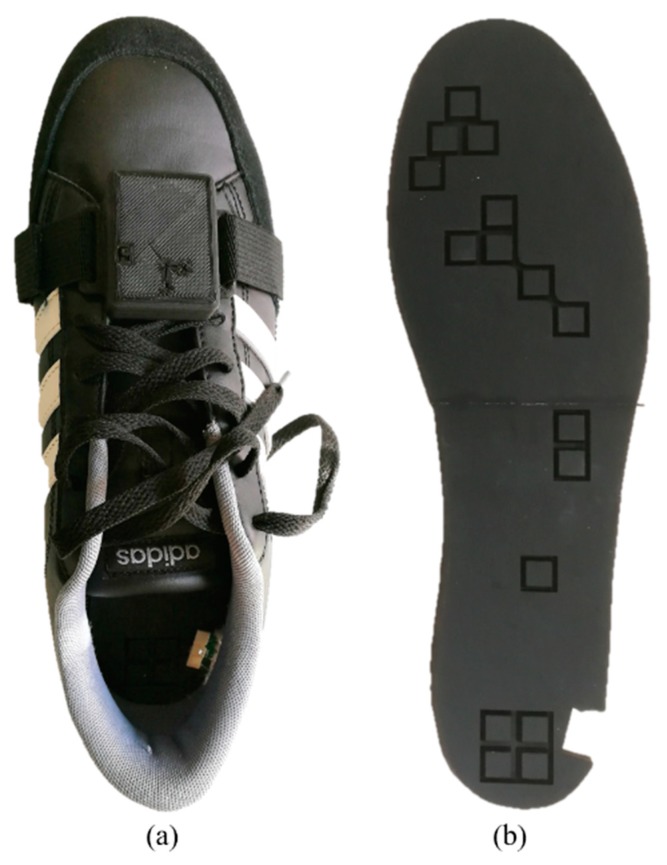
Prototype of pressure-sensitive insoles. (**a**) Shoes endowed with pressure-sensitive insoles. (**b**) Silicone cover.

The pressure-sensitive insoles are made of two main components: a matrix of pressure-sensitive elements inserted in commercial shoes, and on-board electronics for signal conditioning and data transmission that is encased in a lightweight plastic box that can be tied to the shoelaces (Figure 1a). Sensor data are sampled and transmitted at 100 Hz through a wireless Ultra-Wide Band (UWB) protocol (DWM1000, DecaWave 6.8 Mbps data rate) to a portable, remote receiving unit that computes the foot–ground interaction variables through an integrated real-time processor (NI SOM SbRIO-9651, National Instruments^TM^).

The insoles are designed to provide an estimation of the vertical component of the ground reaction force (vGRF) and of its instantaneous point of application along the foot longitudinal axis (i.e., the anterior–posterior coordinate of the center of pressure (CoP_AP_)).

#### 2.1.1. Sensor Technology

Force sensing along the vertical direction was achieved using an optoelectronic technology originally developed to measure human–robot interaction forces [32] and re-engineered for plantar pressure measurements [27]. The force was measured using an array of sensing elements named *tactels*, each one made of a Light-Emitting Diode (LED)-photodiode pair (OSA Opto Light GmbH, Berlin, Germany; Broadcom Ltd., formerly Avago Technologies Ltd., San Jose, CA, USA) coupled to a deformable silicone cover (Figure 1b), which had the shape of a pyramidal frustum with a square base and an internal central curtain. The sensor works as a force-to-voltage transducer: when a load is applied on its top surface, the silicone cover deforms and the curtain gradually closes the light path between the emitter and the receiver, causing a change in the output voltage [27]. The optoelectronic components are soldered on a foot-shaped custom PCB fixed to a 1 mm-thickness carbon lamina that sustains the load while reducing the bending of the insole at push-off.

**Figure 2:**
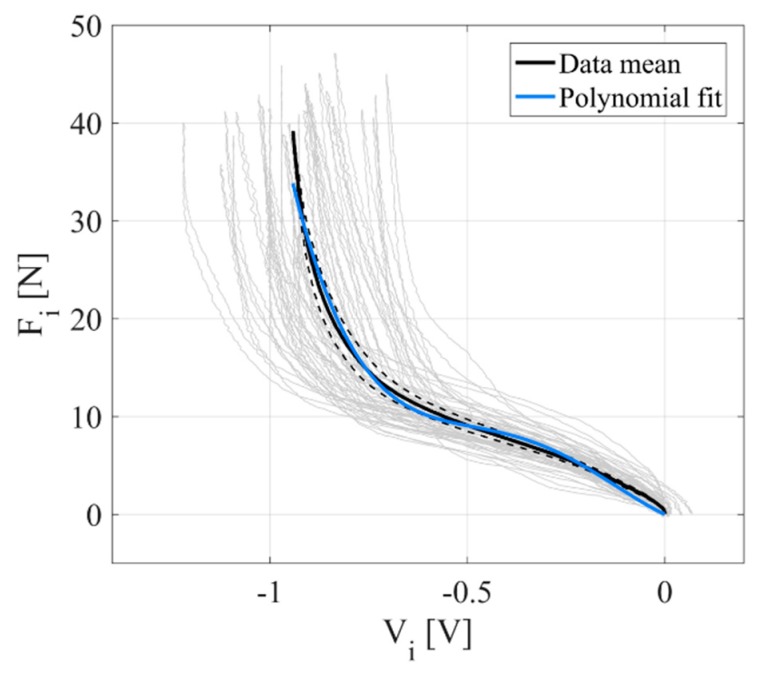
Tactel force-to-voltage relation. Experimental data from 32 sensors (grey); mean load–unload curves (dashed black) and overall mean curve (solid black); polynomial fit of the overall mean curve (blue).

The tactel’s characteristic force-to-voltage relation was extracted with the quasi-static load–unload cycles (velocity of the indenter ~0.1 mm/s); the maximum force applied on each tactel was ~40 N, corresponding to the saturation of the output voltage (Figure 2).

A 4th grade polynomial expression was fitted to the experimental mean curve that was obtained by averaging the mean load–unload curves of 32 sensors (two insoles), resulting in relation (1) (Table 1):

(1) $$F=p_{1}V^{4}+p_{2}V^{3}+p_{3}V^{2}+p_{4}V+p_{5}$$

$$p_{1}=186.1$$

$$p_{2}=224.5$$

$$p_{3}=64.76$$

$$p_{4}=-18.59$$

$$p_{5}=0$$

Such an approximation of the actual force-to-voltage relation of each tactel (Figure 2) could lead to significant errors in the force estimation, due to across-sensor variability and sensor viscoelasticity. However, this limitation was not expected to significantly affect the performance for gait-event detection, primarily related to the latency of sensor response.

#### 2.1.2. Biomechanical Variables

The output voltage on each tactel, $V_{i}$, is converted into a force, $F_{i}$, by means of a piecewise force-to-voltage function: if the output voltage is higher than a pre-determined threshold value ($V_{thresh}$, corresponding to the noise voltage in unloaded conditions), the force is null; otherwise the force-to-voltage calibration function is applied.

The vGRF is calculated as the sum of the forces applied to all tactels:

(2) $$vGRF=\overset{16}{\underset{i=1}{\sum }}F_{i}\;F_{i}=\{\begin{array}{c} f\left(V_{i}\right)\;V_{i}\leq V_{thresh}\\ 0\;V_{i}>V_{thresh} \end{array}$$

$$F_{i}=\mathrm{tactel}\;\mathrm{force}\;\left[N\right]$$

$$V_{i}=\mathrm{tactel}\;\mathrm{output}\;\mathrm{voltage}\;\left[V\right]$$

$$V_{thresh}=\mathrm{noise}\;\mathrm{output}\;\mathrm{voltage}\;\mathrm{threshold}\;\left[V\right]$$

A threshold-based algorithm was applied to the vGRF to segment the gait cycle into stance and swing phases and to enable the calculation of the CoP_AP_ only during stance. The CoP_AP_ was computed by weighting the response of each activated sensor by its longitudinal coordinate ($AP_{i}$) and by the tactel spatial density at that coordinate ($w_{AP_{i}}$) to account for the clustered sensor distribution over the plantar surface:

(3) $$CoP_{AP}=\{\begin{array}{c} \frac{\sum_{i=1}^{16}\left(F_{i}\cdot W_{AP_{i}}\cdot AP_{i}\right)}{\sum_{i=1}^{16}\left(F_{i}\cdot W_{AP_{i}}\right)}\;vGRF\geq vGRF_{thresh}\\ NaN\;vGRF<vGRF_{thresh} \end{array}$$

$$F_{i}=\mathrm{tactel}\;\mathrm{force}\;\left[N\right]$$

$$AP_{i}=\mathrm{tactel}\;\mathrm{antero}-\mathrm{posterior}\;\mathrm{coordinate}\;\left[\mathrm{cm}\right]$$

$$w_{AP_{i}}=\mathrm{tactel}\;\mathrm{antero}-\mathrm{posterior}\;\mathrm{coordinate}\;\mathrm{weight}\;\left[\#\right]$$

$$vGRF_{thresh}=\mathrm{foot}-\mathrm{contact}\;\mathrm{threshold}\;\left[N\right]$$

#### 2.1.3. Sensor Placement

Insoles sensorized with 9–12 sensors have been proven to provide appropriate force measurements [33,34]. In [4], 15 sensors covering the corresponding anatomical areas identified in [35] have been proposed as an optimal sensor configuration. Based on this evidence and considering the practical design requirements of the acquisition electronics, the novel insole was designed to integrate 16 sensors distributed over the plantar surface in relevant locations for monitoring the foot–ground interaction force.

**Figure 3:**
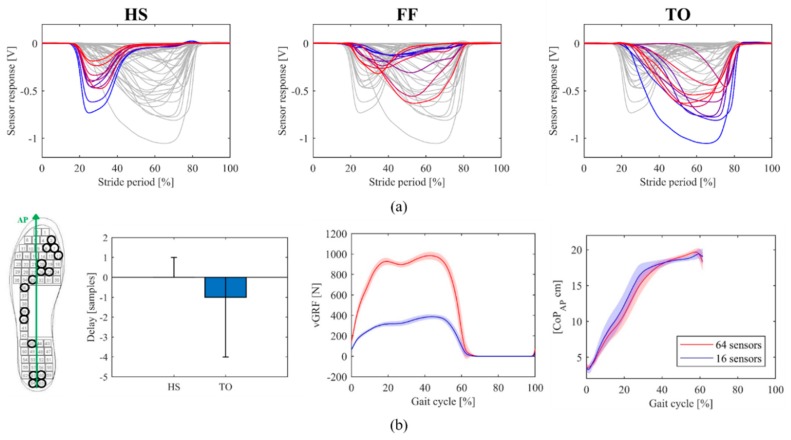
(**a**) Selection of the 10 most relevant sensors for each gait event for a sample subject. (**b**) Sensor placement and offline performance of the configuration with 16 sensors with respect to the one with 64.

To identify the optimal sensor locations, we collected gait data from six healthy subjects walking with the previous prototype of pressure-sensitive insoles featuring 64 sensors distributed over the whole plantar surface [27]. Subjects walked on a treadmill at self-selected speed for about 10 min. Offline data processing was aimed at selecting three subsets of ten sensors for the recognition of the heel-strike (HS), foot-flat (FF), and toe-off (TO) gait events. *Responsivity* and *amplitude* of each sensor signal were considered primary parameters to rank the sensors (Figure 3a). Then, the six different sets of 30 sensors identified for each subject were used to define five different combinations of 16 sensors. This process was carried out by visual inspection, picking the most recurring locations across subjects for each gait event. Finally, the optimal set of sensors was selected as the one displaying the best performance (i.e., synchrony) in detecting the HS and TO events, compared to the 64-sensor configuration (Figure 3b). Offline analysis showed that in 90% of the recorded strides, the selected combination of sensors allowed the detection of the HS event and the TO event, respectively, without delay and one sample (i.e., 10 ms) in advance with respect to the 64-sensor configuration (Figure 3b).

The final sensor configuration covers the foot areas exhibiting the highest plantar pressure concentrations during gait [36] and looks similar to other in-shoe prototypes with a limited number of pressure sensors [33,35]. Naturally, the reduction in the number of sensors resulted in a lower amplitude of the total measured vGRF, whereas the profiles of the CoP did not appear to be significantly affected (Figure 3b).

### 2.2. Verification of the Biomechanical Variables

The insole’s capability to detect gait events and extract temporal parameters was assessed using a commercial force platform. Experiments were carried out in a motion tracking laboratory equipped with two force plates and six infrared cameras.

#### 2.2.1. Experimental Protocol

Two reflective markers were positioned on the tip (toe) and the heel of the shoes equipped with the sensorized insoles to identify the anterior–posterior foot axis. Marker trajectories were recorded with an optical motion capture system (BTS Bioengineering, Italy) at 100 Hz. The same system acquired synchronous data from two adjacent force platforms (BTS Bioengineering, Italy) at 200 Hz. An external analog trigger was set for the temporal alignment of the recorded motion tracking data (including markers and force plate outputs) with the insoles data.

Ten healthy subjects (eight males, 28.6 ± 5.1 years, 174.0 ± 3.8 cm, 66.2 ± 7.1 kg, 21.9 ± 2.40 Body Mass Index (BMI)), without any reported gait disorder or awareness of specific gait deviations or cognitive disorders and with foot sizes in the range 41–43 EU, were recruited for the experiment. All subjects gave their informed consent for inclusion before they participated in the study. The study was conducted in accordance with the Declaration of Helsinki, and the protocol was approved by the local Ethics Committee (Ethics Committee of Area Vasta Centro Toscana, approval number: 12739_spe). Subjects were asked to walk wearing the sensorized shoes along a 10-m walkway at two different speeds—first at a self-selected, comfortable speed (*self*) and then at a self-selected, slow pace (*slow*)—and were instructed to step over two force plates along the way. The force plates were aligned along the forward direction, one next to the other. Small step adjustments before hitting the force plates were permitted, in order to take a clean step on each platform and thus record two consecutive steps, i.e., one with each foot, for each walkway. Targeting was not considered as an issue, since the main goal of the experiment was to compare the force profiles of two devices rather than to use those profiles for assessing gait biomechanics. For each velocity, 20 walkway passes were recorded.

Prior to the start of each speed condition, the output voltage of the sensors was de-offset by the “zero-load” value (i.e., the voltage value acquired when the subject had his/her foot lifted and thus approximately no load was being applied on the sensors).

#### 2.2.2. Data Analysis

Offline data analysis was carried out in MATLAB (MathWorks, Inc., Natick, MA, USA). For each subject, the data collected from different instrumentation systems (insoles vs. motion capture + force plates) were temporally aligned and segmented into single strides. Only the steps recorded on the force platforms were included in the analysis.

For the considered steps, the vGRF and the CoP_AP_ were computed, namely the vGRF_In_ and CoP_In_ for the insoles and the vGRF_Fp_, CoP_AP_Fp_ for the force plates. Notably, the CoP_AP_Fp_ was computed along the foot’s anterior–posterior direction (i.e., the direction determined by the heel and toe markers) to be comparable with the insole CoP_AP_.

To identify the HS and TO events for the force platform, the threshold of the vGRF (vGRF_Thresh_) was set to 20 N, as informally recommended by manufacturer’s representatives and previously adopted with the same system [37]. For the insoles, the vGRF_Thresh_ was set to 3 N (i.e., the force corresponding to all the sensors reading the estimated noise amplitude ($V_{thresh}$)):

(4) $$\{\begin{array}{c} vGRF_{thresh}=16\cdot f\left(V_{thresh}\right)\\ V_{thresh}=-0.01V \end{array}$$

For one subject, the force threshold of the insoles was raised to 7 N during offline analysis to ensure a more precise gait event detection. Several stride parameters were computed to estimate the responsivity of the insoles as well as the quality and the repeatability of the vGRF and the CoPAP profiles. The median values of all of these parameters were computed on all the collected strides as well as separately for the strides collected at each speed. Non-parametric statistics was adopted, as normality tests failed to prove normal distributions for the computed parameters (Lilliefors test, α = 0.05). Right and left insole data were aggregated after verifying that there were no objective differences across the two sides, which were evaluated comparing the right and left stance durations measured by the force plates (Wilcoxon rank sum, *p* = 0.91). Relevant differences across the slow and self-speeds were tested with non-parametric *t*-tests (α = 0.01).

Insole accuracy for the detection of gait events was evaluated estimating the median absolute error (MAE, [s]) for the recognition of the HS, TO, and stance duration with respect to the detection of the same events by the force platform [27], according to Equation (5):

(5) $$MAE_{X}=median\left(\left|X_{Fp}-X_{In}\right|\right)$$

X=HS, TO or stance duration

$$X_{Fp}=X\;\mathrm{time}\;\mathrm{measured}\;\mathrm{by}\;\mathrm{force}\;\mathrm{platform}$$

$$X_{In}=X\;\mathrm{time}\;\mathrm{measured}\;\mathrm{by}\;\mathrm{insoles}$$

The MAEs were also expressed as a percentage of the stance duration measured by the force platform for a comparison across different speeds.

The vGRF peak amplitude (vGRF_peak_) measured by each device was extracted as the maximum value recorded for each step, normalized by the gravitational acceleration (*g*) and expressed as body mass percentage (BM%), in order to make the values comparable across the subjects.

In order to evaluate the consistency of the insole signals with physiological vGRF and CoP_AP_ profiles, the Pearson correlation coefficient (ρ_CoP_, ρ_vGRF_) and the root mean square error (RMSE_CoP_) were computed between the insole and force platform trajectories of the stance phases [27,33,38] (for the vGRF, the RMSE was not computed given the considerable difference in the amplitude of the signals recorded by the two devices). For these calculations, the collected signals were first low-pass filtered and then segmented and interpolated to align the HS and TO events.

The RMSE was also used to investigate measurement repeatability across different steps, computing the error between the single stride and average vGRF and CoP profiles separately for each device (RMSE_CoP_In_, RMSE_CoP_Fp_, RMSE_vGRF_In_, RMSE_vGRF_Fp_). For the vGRF, to account for the different amplitude of the vGRF recorded by the insoles and the force platform, the RMSE was computed using the vGRF normalized by the body mass and then normalized by the stride mean vGRF.

## 3. Results

The analysis included 744 steps, accounting for 91% of those recorded. The remaining trials—on average, five for each subject—were discarded due to an incorrect foot placement on the force platforms or an invalid recording procedure for the alignment of the datasets of the two devices.

**Figure 4:**
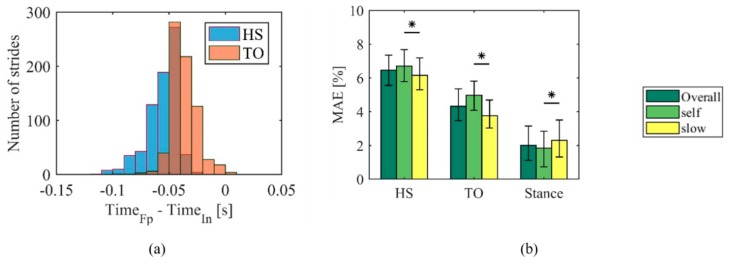
(**a**) Histogram distribution of the difference between the instants of force platform and insole gait-event detection for all the analyzed strides. Blue bars refer to heel-strike (HS) events; orange ones to toe-off (TO). Bar width was set equal to the sampling time. (**b**) Median Absolute Error (MAE) for the HS and TO events and for stance duration, expressed as percentage of the stance duration. The MAE was computed over (i) all the recorded strides (Overall), the strides at self-selected speed (self), and the strides at slow speed (slow).

Figure 4a displays the distribution of the temporal difference between the HS and TO detection performed by the force platform and the insoles. Negative values indicate delayed event recognitions by the insoles compared to the force platform. This result, combined with the higher absolute values for the HS than the TO, resulted in an overall shorter stance duration recorded with the insoles.

The overall insole MAE was 0.06 (0.02) s for the HS; 0.04 (0.02) s for the TO; and 0.02 (0.01) s for stance duration, corresponding to 6.47 (1.79) %, 4.32 (1.89) % and 2.02 (2.03) % of the stance duration, respectively (Figure 4b).

Considering the *slow* and *self* data separately, significant differences existed in the percent MAE of the HS, TO, and stance duration, with faster event recognition at slower speeds.

**Figure 5:**
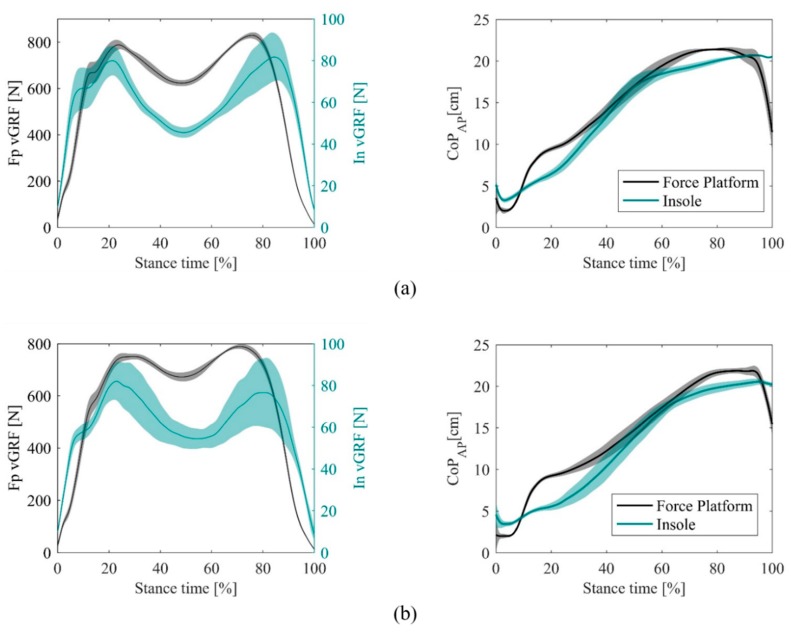
Average (right) vGRF and CoP_AP_ stance curves measured by the force platform (black) and insole (green) for one participant during the self-selected (**a**) and slow (**b**) speed trials.

Concerning the temporal profiles of the biomechanical signals, Figure 5 compares a participant’s average (right) vGRF and CoP_AP_ curves measured by the two devices during the corresponding stance phase. The amplitude of the vGRF measured by the insoles was smaller than the physiological (force-platform) one, with maximum peaks of 19.92 (8.60) BM%, that is 19% of the corresponding force platform measure (Table 2). The Pearson correlation coefficient between the average vGRF profiles of the two devices was 0.47(0.20) (Table 2). For CoP_AP_ profiles, the consistency between the trajectories of the two devices was higher, with a ρ_CoP_ of 0.96 (0.02), while the RMSE_CoP_ was 2.29 (0.58) cm (Table 3). The effect of the speed was visible on vGRF profiles, which exhibited less pronounced “M” shapes with both devices at lower speeds. At slower speeds, the correlation between the force platform and insole profiles was lower for the vGRF, but not for the CoP_AP_, whose RMSE also decreased.

For measurement repeatability, the insole-based measures were less repeatable than the force platform for the estimation of the vGRF, as demonstrated by the higher standard deviation bands in the profiles of Figure 5 and the higher values of the RMSE_vGRF_In_ than that of the RMSE_vGRF_Fp_, which were equal to 13.25 (8.07) % and 4.04 (2.59) % of their mean values, respectively (Table 2). For the CoP_AP_, the measurement repeatability of the devices was similar and in both cases lower than 1 cm, with RMSE_CoP_In_ equal to 0.98 (0.87) cm and RMSE_CoP_Fp_ equal to 0.90 (0.59) cm (Table 3). Slower speeds also decreased the repeatability of insole measurements, as visible when comparing the profiles of Figure 5a,b and indicated by higher RMSE_vGRF_In_ and RMSE_CoP_In_, while the same parameters were not significantly different for the force platform.

## 4. Discussion

### 4.1. Gait Event Recognition

HS and TO event detection occurred later with the insoles than with the force platform. The most pronounced delay was reported for the HS, with a temporal value corresponding to six samples at a sampling rate of 100 Hz. Considering an average stance phase duration of approximately 60% of the stride time [39], the delays for HS and TO recognitions corresponded to 3.9% and 2.6% of the stride period, and the stance duration measurement error was 1.3% of the whole gait cycle.

Compared to the values reported in literature—where only a few systems based on force-sensitive resistors have been characterized for timing performance—our system accurately estimated the stance duration, with good responsiveness in HS and TO recognition, in line with the results reported in previous studies. In the first case, previous studies demonstrated that the Medilogic^®^ insoles could estimate the stance duration with less than 10% error [40], while a system based on two footswitches performed the estimation with a ± 3% error [41]. Regarding gait-events recognition, the reported errors for in-shoe systems compared to force plates fell within 30 ms. In particular, the aforementioned system based on two footswitches had a latency of ± 10 ms for the HS and ± 22 ms for the TO [41], even though the system was tethered—and thus exhibited no latency due to wireless communication—and furthermore could not provide any information on the gait dynamics based on plantar pressure variables. The “GaitShoe” system, relying on force-sensitive resistors for the estimation of gait events and foot-pressure, was found to have −6.7 ± 22.9 ms error for HS recognition and −2.9 ± 16.9 ms for the TO, with respect to a force plate [42]. Though the system was wireless, the impact of the communication latency was not quantified, as the trials with more than 10 missing packets were discarded from the analysis. Finally, the Tekscan F-scan insoles showed delays in the range of 20–30 ms in the time instants of the vGRF local peaks of the data collected by the insoles compared to the data of a force platform. The two datasets had been aligned offline at the instant of detected ground contact [43,44], possibly indicating a similar delay for HS recognition. Thus, considering the results reported for the other insole-based devices, our insoles seemed to be slower in the recognition of foot-contact events, but still timely for online control purposes. In fact, studies validating gait-segmentation algorithms based on inertial sensors have deemed latencies of up to 150 ms acceptable for online functional electrical stimulation [45,46]. According to [10], delays in the range of a few tenths of milliseconds would also be acceptable for assisting locomotion with active exoskeletons as they are lower than reaction time of voluntary muscles (180 ms).

Several factors may have contributed to the delayed HS detection by our system: wireless communication, given the implementation of a UWB (Ultra-Wide Band) protocol within a real-time loop operating at 100 Hz, can account for a lag of 10 ms (i.e., one sample). Then, the recognition delays observed with our system may be further ascribable to the suboptimal conversion of the raw voltage signals of the sensors into force. In fact, the adopted force–voltage relation was obtained with mathematical approximations that did not fully account for the mechanical behavior of the sensor under dynamic operating conditions. Indeed, the silicone membrane confers the sensor with typical polymeric properties such as viscoelasticity, damping, and hysteresis, the latter also being visible from the characterization curves of Figure 2. At the same time, as a result of having been extracted via a polynomial fit to the quasi-static loading profile over the operational force range, the adopted force–voltage equation could have underestimated the actual force when the output signals are low and when the load is applied very fast such as at the HS. The tendency toward lower errors for slower speeds would support viscoelasticity as an explanation for a part of the delay in instances of fast loading/unloading. Silicone hysteresis on the other hand, could explain different delays between the HS and the TO.

### 4.2. Profiles of Biomechanical Signals

Considering the vGRF temporal profiles, the signals recorded by the insoles had considerably smaller amplitude than recordings from the force platform, a direct consequence of the limited sensorized plantar area that resulted from reducing the number of sensors of the insole. This choice also affected the quality of the estimated temporal profile: correlation indices indicated only a “low” degree of association [47] in contrast to the “high” grade achieved by the former prototype [27], and below the average results for most of the available in-shoe devices, which rank from “high” to “very high” [20,33,38].

Insole performance in estimating the vGRF profile is directly related to sensor distribution: though the sensor locations visually match the areas subject to the highest plantar pressures during gait, the limited number and non-uniform distribution of sensors significantly altered the final vGRF profile. In [1], Park et al., who developed another opto-electronic system with four discrete sensors, improved the consistency with force plate measurements by assigning specific weights to the contribution of each sensor to fit the overall vGRF, instead of simply adding up the signals. However, this scaling calculation requires subject- and task-specific calibration procedures that are impractical to repeat in the desired clinical and community use scenarios.

In our case, the configuration was not optimized for vGRF measurement accuracy but for gait event recognition accuracy. From this perspective, the clustered sensor distribution allowed the placement of multiple redundant sensors in the most functionally relevant positions for maintaining reliable performance in the case of sensor failure, in accordance with the primary intended utilization of the insoles as accurate gait phase estimators rather than precise gait analysis tools.

Nevertheless, the insoles were able to provide consistent CoP_AP_ estimates with successful compensation for the non-uniform sensor distribution via weighting each sensor’s contribution by the number of sensors located at the same coordinate. The CoP_AP_ profiles showed “very high” correlation and only moderate error relative to the force plate at both tested speeds, with outcomes comparable with the other existing devices [20,33,38]. This result was especially relevant considering the limited accuracy of the experimental setup for this calculation and the 2D approximation in referring the CoP estimated by the force platform to the insole local coordinate system.

Regarding measurement repeatability, the variability of the vGRF profiles was markedly higher for those recorded by the insoles than by the force-plate, while their respective performances were similar for the estimation of the CoP_AP_. The higher insole variability for the vGRF profiles was probably another consequence of the partially sensorized plantar area. Indeed, the insoles may overestimate the actual variability of the vGRF because relatively small variations in pressure distribution between the sensitive and non-sensitive areas across strides may result in markedly different vGRF profiles.

## 5. Conclusions

In this paper, we presented a simplified prototype of the pressure-sensitive insoles described in [27], optimized for gait-events recognition with a reduced number of sensors, with the objective of enabling real-time applications such as with active orthoses, prostheses, and sensory feedback.

The results of the experimental verification against a commercial force platform showed that the new system was capable of recognizing the HS and TO events with approximately 60 ms and 40 ms of delay (respectively), and of estimating the stance duration with an error of 1.3% of the stride period. The insoles proved accurate for the estimation of the duration of the stance/swing phases compared to the other existing, benchmark portable devices. The estimated delays in the recognition of the HS and TO events may be suitable for the intended use case of this device (i.e., gait phase estimation in the context of wearable robotics). Moreover, the observed trend toward smaller errors for slower walking speeds could work favorably for the insoles, which were primarily intended for users with limited ambulation abilities.

Nonetheless, additional approaches for gait-event recognition shall be investigated to improve temporal accuracy such as the application of threshold-based algorithms directly to the raw voltage signals of the heel sensors, or the modification of the force-to-voltage relation to increase the sensitivity at low voltages. Notably, such an approach could further bias the estimation of the vGRF. Another solution could consist of introducing different force thresholds for the HS and TO events to account for hysteresis and viscoelasticity in the sensor response.

In any case, optoelectronic transduction seemed less responsive than force resistors, but it could not be ranked in relation to other sensing technologies as, to the authors’ knowledge, no reference data are available for most of the existing systems. Moreover, other design aspects related to sensor technology limit the adaptability of the current prototype to the foot sizes and gait patterns of different users. In the first case, though the sensor configuration could be easily scaled, tactel dimensions would remain unvaried, thus determining a relatively larger/smaller sensitive area for smaller/larger foot sizes. Regarding adaptability to different gait patterns, in case of users with pathological gait, insole performance could further vary on a subject basis, as the optimized sensor configuration was obtained elaborating the data of subjects without known abnormal gait patterns. Depending on the impact of these deviations on the estimation of insole signals, more advanced data processing could be required to maintain reliable gait-events recognition and estimation of vGRF and CoP profiles.

Reducing the pressure-sensitive area of the current insole design with respect to the previous prototype has reduced the insole performance for the estimation of the vGRF. Given the low degree of correlation with the profiles recorded by the force platform, the vGRF estimated by the insoles would not constitute an appropriate variable for quantitative gait analysis. On the other hand, the estimation of the CoP_AP_ was demonstrated to be reliable and on par with the accuracy of the other existing in-shoe devices. Therefore, besides the primary intended use case as sensors for the control of robotic devices, the insoles could be contextually exploited for gait assessments based on monitoring the CoP_AP_ and the main temporal parameters, given the fair accuracy in the quantification of the stance phase duration. Furthermore, it may still be possible to improve the insole-based estimate of vGRF by introducing subject-specific calibration procedures similarly to [1], though the quality of the vGRF signals is bound to remain limited by the partial sensitive area. Finally, a complimentary consideration on the comparison of the two versions relates to the costs of the two prototypes. The novel insole has a significantly lower number of sensory components (75%) and in turn, a significantly simpler electronics for sensor readings and communication. Such practical advantages may influence the potential use of the technology in non-medical applications, where reliable temporal information is sufficient to provide relevant gait information and cost represents a limiting factor for the adoption. Still, given the easily scalable sensing technology, a different sensing configuration featuring a completely sensorized plantar area would be preferable for gait assessment scenarios in which vGRF is a variable of interest and cost does not represent a limiting factor for its adoption.

## Figures and Tables

**Table 1:** Goodness of fit of Equation (1).

Fit Type	SSE	R-Square	Adjusted R-Square	RMSE
Polynomial, 4th grade	544	0.9898	0.9897	0.7391

**Table 2:** Median (iqr) values for the vertical Ground-Reaction Force (vGRF) parameters.

	Insole		Force Platform		Comparison
	vGRF_peak_In_ [% BM]	RMSE_vGRF_In_ [%]	vGRF_peak_Fp_ [% BM]	RMSE_vGRF_Fp_ [%]	ρ_vGRF_ [#]
**Overall**	19.92 (8.60)	13.25 (8.07)	106.58 (8.40)	4.04 (2.59)	0.47 (0.20)
**self**	21.80 (8.77)	12.26 (7.55)	110.27 (9.27)	3.99 (2.58)	0.48 (0.19)
**slow**	18.56 (7.55)	14.40 (8.82)	103.79 (4.3)	4.08 (2.61)	0.45 (0.22)

**Table 3:** Median (iqr) values for the antero-posterior coordinate of the Center of Pressure (CoP_AP_) parameters.

	Insole	Force Platform	Comparison	
	RMSE_CoP_In_ [cm]	RMSE_CoP_Fp_ [cm]	ρ_CoP_ [#]	RMSE_CoP_ [cm]
**Overall**	0.98 (0.87)	0.90 (0.59)	0.96 (0.02)	2.29 (0.58)
**self**	0.82 (0.79)	0.90 (0.62)	0.96 (0.02)	2.39 (0.64)
**slow**	1.16 (0.86)	0.90 (0.56)	0.97 (0.01)	2.22 (0.53)

## References

[1] Park J., Na Y., Gu G., Kim J. Flexible insole ground reaction force measurement shoes for jumping and running. Proceedings of the IEEE RAS and EMBS International Conference on Biomedical Robotics and Biomechatronics.

[2] Ramirez-Bautista J.A., Huerta-Ruelas J.A., Chaparro-Cárdenas S.L., Hernández-Zavala A. (2017). A review in detection and monitoring gait disorders using in-shoe plantar measurement systems. IEEE Rev. Biomed. Eng..

[3] Zhang H., Zanotto D., Agrawal S.K. (2017). Estimating CoP Trajectories and Kinematic Gait Parameters in Walking and Running Using Instrumented Insoles. IEEE Robot. Autom. Lett..

[4] Abdul Razak A.H., Zayegh A., Begg R.K., Wahab Y. (2012). Foot plantar pressure measurement system: A review. Sensors (Switzerland).

[5] Maqbool H.F., Husman M.A.B., Awad M.I., Abouhossein A., Iqbal N., Dehghani-Sanij A.A. (2017). A Real-Time Gait Event Detection for Lower Limb Prosthesis Control and Evaluation. IEEE Trans. Neural Syst. Rehabil. Eng..

[6] Novak D., Riener R. (2015). A survey of sensor fusion methods in wearable robotics. Rob. Auton. Syst..

[7] González I., Fontecha J., Hervás R., Bravo J. (2015). An ambulatory system for gait monitoring based on wireless sensorized insoles. Sensors.

[8] Afzal M.R., Oh M.K., Lee C.H., Park Y.S., Yoon J. (2015). A portable gait asymmetry rehabilitation system for individuals with stroke using a vibrotactile feedback. Biomed. Res. Int..

[9] Chen B., Wang X., Huang Y., Wei K., Wang Q. (2015). A foot-wearable interface for locomotion mode recognition based on discrete contact force distribution. Mechatronics.

[10] Figueiredo J., Moreno J.C., Santos C.P. Assistive locomotion strategies for active lower limb devices. Proceedings of the ENBENG 2017—5th Portuguese Meeting on Bioengineering.

[11] Zheng H., Yang M., Wang H., Mcclean S. (2009). Machine learning and statistical approaches to support the discrimination of neuro-degenerative diseases based on gait analysis. Stud. Comput. Intell..

[12] Hegde N., Bries M., Sazonov E. (2016). A comparative review of footwear-based wearable systems. Electronics.

[13] Chen B., Papapicco V., Parri A., Crea S., Munih M., Vitiello N. (2019). A Preliminary Study on Locomotion Mode Recognition with Wearable Sensors. Biosystems and Biorobotics.

[14] Tucker M.R., Olivier J., Pagel A., Bleuler H., Bouri M., Lambercy O. (2015). Control strategies for active lower extremity prosthetics and orthotics : A review. J. NeuroEng. Rehabil..

[15] Jiménez-Fabián R., Verlinden O. (2012). Review of control algorithms for robotic ankle systems in lower-limb orthoses, prostheses, and exoskeletons. Med. Eng. Phys..

[16] Yuan K., Sun S., Wang Z., Wang Q., Wang L. A fuzzy logic based terrain identification approach to prosthesis control using multi-sensor fusion. Proceedings of the IEEE International Conference on Robotics and Automation.

[17] Lee G., Kim J., Panizzolo F.A., Zhou Y.M., Baker L.M., Galiana I., Malcolm P., Walsh C.J. (2017). Reducing the metabolic cost of running with a tethered soft exosuit. Sci. Robot..

[18] Beravs T., Rebersek P., Novak D., Podobnik J., Munih M. Development and validation of a wearable inertial measurement system for use with lower limb exoskeletons. Proceedings of the 2011 11th IEEE-RAS International Conference on Humanoid Robots.

[19] Tao W., Liu T., Zheng R., Feng H. (2012). Gait Analysis Using Wearable Sensors. Sensors.

[20] Dyer P.S., Bamberg S.J.M. Instrumented insole vs. force plate: A comparison of center of plantar pressure. Proceedings of the Annual International Conference of the IEEE Engineering in Medicine and Biology Society.

[21] Luo Z.P., Berglund L.J., An K.N. (1998). Validation of F-Scan pressure sensor system: A technical note. J. Rehabil Res. Dev..

[22] Price C., Parker D., Nester C. (2016). Validity and repeatability of three in-shoe pressure measurement systems. Gait Posture.

[23] Woodburn J., Helliwell P.S. (1996). Observations on the F-Scan in-shoe pressure measuring system. Clin. Biomech..

[24] Tan A.M., Fuss F.K., Weizman Y., Woudstra Y., Troynikov O. (2015). Design of low cost smart insole for real time measurement of plantar pressure. Procedia Technol..

[25] Morris S.J. (2004). A Shoe-Integrated Sensor System for Wireless Gait Analysis and Real-Time Therapeutic Feedback. Ph.D. Thesis.

[26] Varoto R., Oliveira G.C., De Lima A.V.F., Critter M.M., Alberto Cliquet A. A low cost wireless system to monitor plantar pressure using insole sensor: Feasibility approach. Proceedings of the BIODEVICES 2017—10th International Conference on Biomedical Electronics and Devices.

[27] Crea S., Donati M., De Rossi S., Oddo C., Vitiello N., Crea S., Donati M., De Rossi S.M.M., Oddo C.M., Vitiello N. (2014). A wireless flexible sensorized insole for gait analysis. Sensors.

[28] Crea S., Cipriani C., Donati M., Carrozza M.C., Vitiello N. (2015). Providing time-discrete gait information by wearable feedback apparatus for lower-limb amputees: Usability and functional validation. IEEE Trans. Neural Syst. Rehabil. Eng..

[29] Crea S., Edin B.B., Knaepen K., Meeusen R., Vitiello N. (2017). Time-discrete vibrotactile feedback contributes to improved gait symmetry in patients with lower limb amputations: Case series. Phys. Ther..

[30] Martini E., Baldoni A., Fiumalbi T., Dell’Agnello F., Crea S., Vitiello N. Metodo per la disposizione ottimizzata di sensori di pressione e dispositivo ottenuto con tale metodo 2019.

[31] De Rossi S.M.M., Lenzi T., Vitiello N., Persichetti A., Giovacchini F., Carrozza M.C. Structure of sensorized mat 2011.

[32] Donati M., Vitiello N., de Rossi S.M.M., Lenzi T., Crea S., Persichetti A., Giovacchini F., Koopman B., Podobnik J., Munih M. (2013). A flexible sensor technology for the distributed measurement of interaction pressure. Sensors (Switzerland).

[33] Claverie L., Ille A., Moretto P. (2016). Discrete sensors distribution for accurate plantar pressure analyses. Med. Eng. Phys..

[34] Howell A.M., Kobayashi T., Hayes H.A., Foreman K.B., Bamberg S.J.M. (2013). Kinetic gait analysis using a low-cost insole. IEEE Trans. Biomed. Eng..

[35] Shu L., Hua T., Wang Y., Li Q., Feng D.D., Tao X. (2010). In-shoe plantar pressure measurement and analysis system based on fabric pressure sensing array. IEEE Trans. Inf. Technol. Biomed..

[36] Hessert M.J., Vyas M., Leach J., Hu K., Lipsitz L.A., Novak V. (2005). Foot pressure distribution during walking in young and old adults. BMC Geriatr..

[37] Abolins V., Bernans E., Lanka J. (2018). Differences in vertical ground reaction forces during first attempt of barefoot running in habitual shod runners. J. Phys. Educ. Sport.

[38] Chesnin K.J., Selby-Silverstein L., Besser M.P. (2000). Comparison of an in-shoe pressure measurement device to a force plate: Concurrent validity of center of pressure measurements. Gait Posture.

[39] Borghese N.A., Bianchi L., Lacquaniti F. (1996). Kinematic determinants of human locomotion. J. Physiol..

[40] DeBerardinis J., Dufek J.S., Trabia M.B., Lidstone D.E. (2018). Assessing the validity of pressure-measuring insoles in quantifying gait variables. J. Rehabil. Assist. Technol. Eng..

[41] Hausdorff J.M., Ladin Z., Weis J.Y. (1995). Footswitch system for measurement of the temporal parameters of gait. J. Biomech..

[42] Bamberg S.J.M., Benbasat A.Y., Scarborough D.M., Krebs D.E., Paradiso J.A. (2008). Gait analysis using a shoe-integrated wireless sensor system. IEEE Trans. Inf. Technol. Biomed..

[43] Chevalier T.L., Hodgins H., Chockalingam N. (2010). Plantar pressure measurements using an in-shoe system and a pressure platform : A comparison. Gait Posture.

[44] Chen B., Bates B.T. (2000). Comparison of F-Scan in-sole and AMTI forceplate system in measuring vertical ground reaction force during gait. Physiother. Theory Pract..

[45] Mansfield A., Lyons G.M. (2003). The use of accelerometry to detect heel contact events for use as a sensor in FES assisted walking. Med. Eng. Phys..

[46] Mannini A., Genovese V., Sabatini A.M. (2014). Online decoding of hidden markov models for gait event detection using foot-mounted gyroscopes. IEEE J. Biomed. Heal. Inform..

[47] Mukaka M.M. (2012). Statistics Corner: A guide to appropriate use of Correlation coefficient in medical research. Malawi Med. J..

